# SMART v10: three decades of the protein domain annotation resource

**DOI:** 10.1093/nar/gkaf1023

**Published:** 2025-10-08

**Authors:** Ivica Letunic, Peer Bork

**Affiliations:** biobyte solutions GmbH, Bothestr 142, 69126 Heidelberg, Germany; EMBL, Meyerhofstrasse 1, 69117 Heidelberg, Germany; Max Delbrück Centre for Molecular Medicine,13125 Berlin, Germany; Department of Bioinformatics, Biocenter, University of Würzburg, 97070 Würzburg, Germany

## Abstract

SMART (Simple Modular Architecture Research Tool, https://smart.embl.de) is a web-based platform for identifying and annotating protein domains and analyzing domain architectures. SMART version 10 features manually curated models for over 1300 protein domains. Approaching its 30th anniversary, SMART’s user interface has been redesigned from the ground up, leveraging modern web technologies to enhance intuitiveness and usability. SMART’s “Genomic” mode, which annotates proteins from completely sequenced genomes was synchronized with the current release of STRING, and now includes 12 035 species, compared to 5090 in the previous release. Protein and domain annotation pages have been updated with new information sources. Integration with eggNOG provides links to 17.5 million orthologous groups for over 53 million proteins. Additionally, synchronization with the interactive Pathways Explorer version 3 incorporates updated KEGG pathway and orthologous group data, enabling direct visualization on four distinct pathway overview maps.

## Graphical Abstract

**Graphical Abstract:**
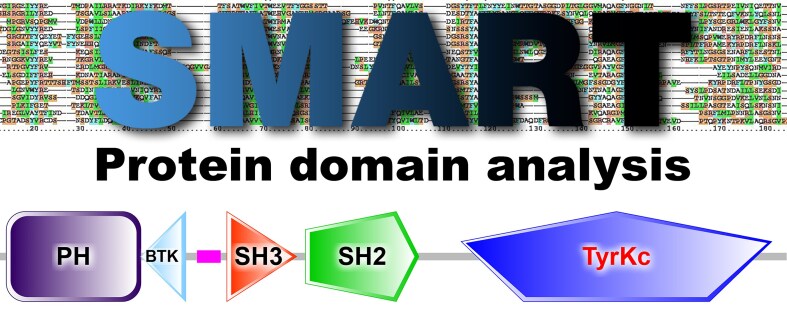

## Introduction

Protein domain analysis remains a crucial research tool, simplified by various user-friendly online databases [1–3] providing comprehensive domain annotations. The SMART database [4] combines manually curated hidden Markov models [5, 6] for many domains with a user-friendly web interface, providing robust analysis and visualization capabilities. For nearly 30 years, SMART has been a widely adopted resource among researchers worldwide.

SMART specializes in mobile protein domains, particularly those involved in signaling, extracellular, and regulatory functions. It emphasizes the modular architecture of proteins, making it particularly valuable for studying complex, multidomain proteins and their evolutionary relationships. SMART’s hidden Markov models are manually curated, ensuring high-quality annotations with minimal false positives. SMART’s web interface is designed for intuitive navigation, offering interactive tools like domain architecture diagrams, phylogenetic distribution views, and detailed annotation tables. These features make it particularly accessible for researchers studying domain evolution and protein function.

Here, we outline the key updates and new features introduced since our last report [7].

## New user interface

**Figure 1.**
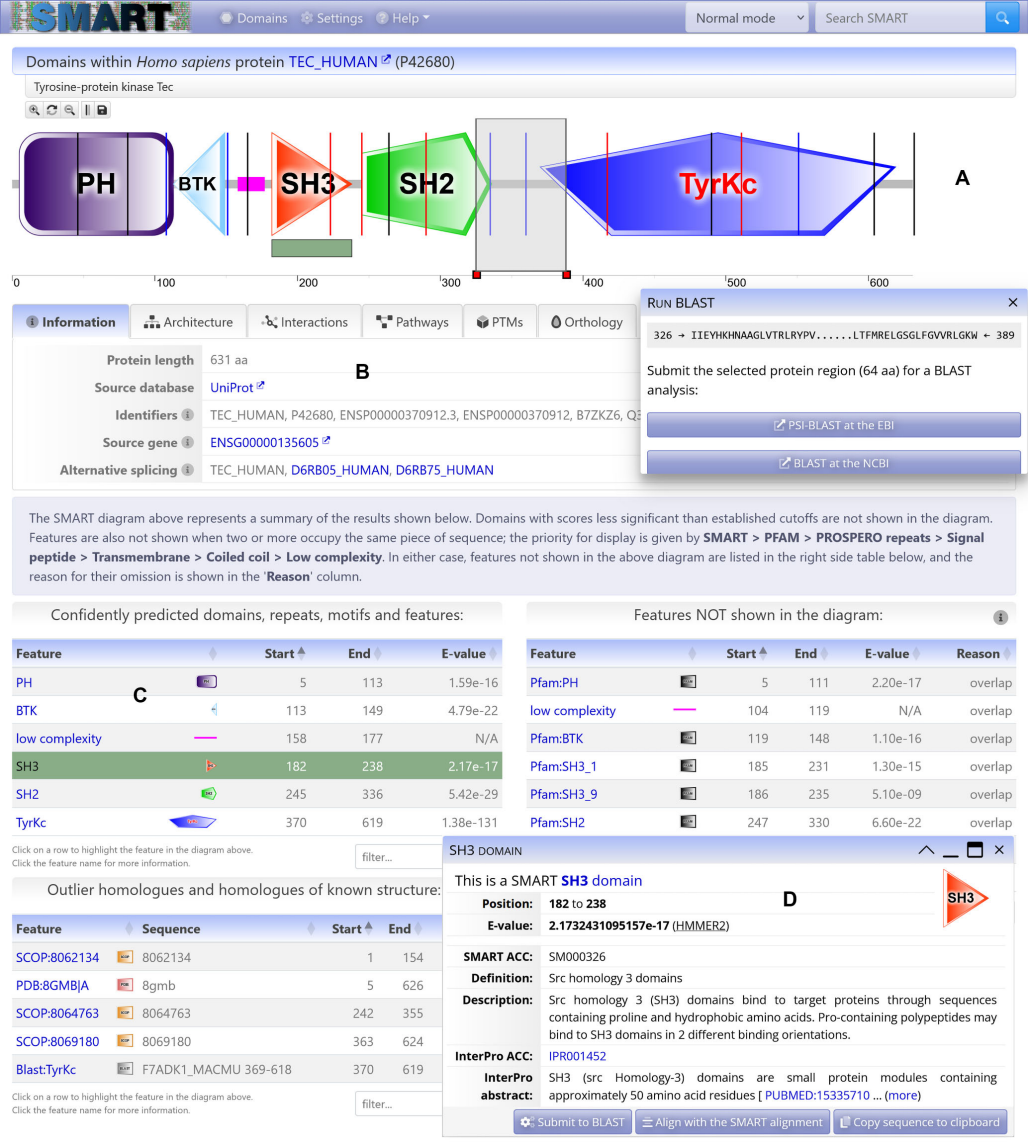
SMART annotation page for protein TEC_HUMAN. (**A**) Protein schematic representations are displayed using an interactive SVG (scalable vector graphics) applet. Schematics are zoomable without quality loss and can be saved as vector (SVG) images. Using the interactive scale, any protein region can be selected and submitted for further BLAST (Basic Local Alignment Search Tool) analysis. (**B**) Tabbed interface collects various sources of external information about the protein analyzed. (**C**) Data tables listing the domains and other features detected in the protein. Each table can be individually searched or sorted on any column. Selecting a feature will highlight it in the protein schematic, while scrolling the display to make it visible on screen. (**D**) Movable and resizable popup dialog displays the most important bits of information for any selected feature, with links to complete annotation.

SMART version 10 introduces a fully redesigned web user interface, rebuilt from the ground up to leverage modern web technologies (Fig. 1). The redesign preserves the original functionality and page layout to ensure a familiar user experience as much as possible.

Protein domain annotation pages now feature a tabbed interface, incorporating a faster, interactive tree display widget for exploring the taxonomic distribution of related proteins.

All protein schematics in list displays, such as domain architecture analysis results, are rendered as dynamically generated SVG images, which seamlessly scale to the users display size, regardless of its resolution. In SMART version 10, these have been extended with several new features and optimized to support latest web standards. Protein size scales are now included in all generated images, allowing easier identification of the positions of individual domains and other features.

The display applet used in the single protein annotation mode has been extended and remains fixed on-screen during page scrolling. Selecting a predicted domain or feature in data tables now automatically highlights its position in the protein schematic and centers it on the screen. This functionality simplifies the identification of relationships among protein features, including those not directly displayed due to threshold cutoffs or overlaps.

If a more fine-grained evaluation of a protein region is required, the viewer allows interactive selection of various parts of the protein sequence independent of the annotated features and their submission to BLAST analysis [8]. This function now supports direct submission of the sequence to the PSI-BLAST [9] server at the European Bioinformatics Institute [10], or to the standard BLAST server at NCBI [11].

## Updated genomic protein database

The main underlying protein database in SMART combines the complete UniProt [12] with all stable Ensembl [13] proteomes. In addition to the main protein database, SMART offers a “Genomic” analysis mode that contains only proteins from completely sequenced genomes. Synchronized with the current STRING version 12 [14], it contains >59 million proteins from 12 535 complete genomes (1322 Eukaryota, 10 756 Bacteria, and 457 Archaea), which is a 2.5-fold increase in both the number of proteins and genomes compared to the previous release.

## Expanded protein interaction data

With the update of the underlying protein databases, we have also synchronized our protein interaction data with the version 12 of the STRING database [14]. Updated graphical representations of putative interaction partners are now available for >52 million proteins. These interaction network displays in SMART are now interactive and can be zoomed and navigated directly. Individual proteins in the network can be selected, and their corresponding annotation pages in SMART or STRING accessed for further exploration.

## Expanded and updated external information sources

SMART version 10 significantly expands its integration of external data sources. Protein orthology data, sourced from the eggNOG 6.0 database [15], now encompass over 53 million proteins across 12 035 species, organized into >17 million orthologous groups. Annotation pages provide a detailed overview of orthologous groups for each protein, including descriptions and taxonomic classifications. Direct links to eggNOG 6.0 enable in-depth exploration of these groups, complete with their associated alignments and phylogenetic trees.

Biological pathway integration has been substantially enhanced, with synchronization to the interactive Pathways Explorer version 3 (iPath) [16]. Pathway data, available for nearly 40 million proteins, now include links to 423 KEGG pathways and 15 758 KEGG orthologous groups (KOs) [17]. Users can visualize these pathways and KOs interactively on four iPath3 overview maps: metabolic pathways, biosynthesis of secondary metabolites, biosynthesis of antibiotics, and microbial metabolism in diverse environments.

## Updated architecture SMART and taxonomic tree data export

SMART’s domain architecture analysis enables users to identify proteins with specific domain combinations. These can also be generated using combinations of GO (Gene Ontology) terms [18] associated with protein domains and restricted to selected taxonomic classes. In addition to the standard SMART protein schematic visualization, these data can also be exported into FASTA files or represented as annotated phylogenetic trees.

**Figure 2.**
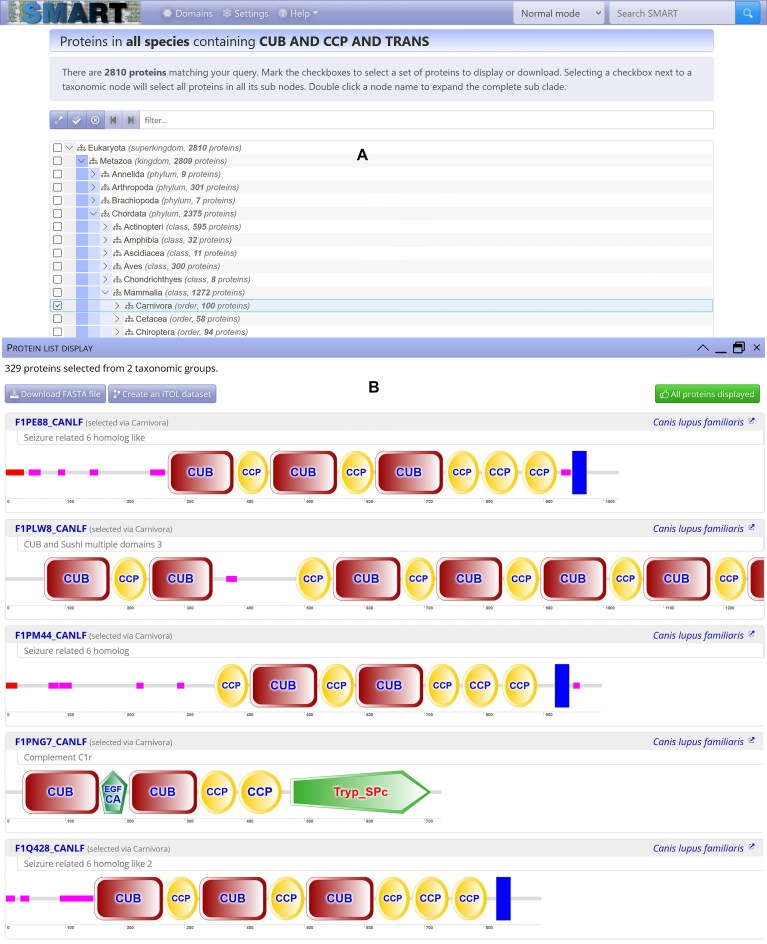
SMART’s new domain architecture analysis page. (**A**) All proteins containing the domains matching user’s query are displayed in a taxonomic tree browser. User can select any combination of taxonomic clades or individual proteins. (**B**) Proteins selected by the user are visualized in a dialog window directly in the results page, allowing simpler comparisons of domain architectures between across taxonomic clades. Proteins selected can also be downloaded as a FASTA file or visualized in iTOL (Interactive Tree of Life) [19].

The backend for domain architecture queries has been completely overhauled, achieving 10- to 100-fold faster performance. Analysis results, including datasets exceeding 50 000 proteins, now load quickly and display efficiently in modern web browsers. These results are visualized as a taxonomic tree structure, and SMART protein schematics are displayed directly in a floating popup dialog for any selected tree clade (Fig. 2). This allows simple exploration of large datasets, and quick comparison of protein domain architectures across taxonomic clades.

Phylogenetic tree export functionality has been enhanced and aligned with the iTOL version 6 [19]. Phylogenetic trees and their associated protein domain datasets can be downloaded or visualized directly in iTOL, and now also include helpful popup information with detailed taxonomic information for all clades and species. Furthermore, the taxonomic database used for the tree generation was synchronized with the current NCBI taxonomy release [20].

## Database and web server optimizations

The backend of SMART is a relational database management system powered by PostgreSQL. It stores annotations for all SMART domains, protein annotations and sequences, taxonomy information, and precalculated analyses for the entire UniProt [12], Ensembl [13], and STRING [14] proteomes. In addition to the predictions of all SMART and Pfam [21] domains, this includes various protein intrinsic features, like signal peptides, transmembrane, and coiled coil regions.

In SMART version 10, we now also store the precalculated results for the detection of remote homologues and homologues of protein structures. These are generated via regular BLAST searches against the latest PDB [22], SCOP [23], and the sequences of all detected SMART domains.

Due to constant growth of annotated features, we are regularly restructuring our backend databases, and optimizing various parts of the server code to ensure a satisfactory user experience. Additionally, we have upgraded the server hardware supporting sequence annotation searches and database queries, incorporating expanded RAM and CPU capacity. These enhancements significantly improve processing speeds for user-submitted proteins and reduce response times.

## Data Availability

SMART profiles and alignments are freely available for academic users via EMBLem (https://www.embl-em.de). Commercial licenses and support are available via biobyte solutions GmbH (https://www.biobyte.de).
